# Evaluation of mental disorder related to colposcopy procedure during the COVID period: A cross-sectional study

**DOI:** 10.1177/17455057241308342

**Published:** 2025-01-18

**Authors:** Ilaria Bochicchio, Martina Catalano, Giovanni Deiana, Giandomenico Roviello, Pasquale Marino, Fabrizia Calenda, Alessandro R Lettini, Francesca Sanseverino

**Affiliations:** 1 Unit of Clinical Psychology, Centro di Riferimento Oncologico della Basilicata (IRCCS-CROB), Rionero in Vulture, Italy; 2 Section of Clinical Pharmacology and Oncology, Department of Health Sciences, University of Florence, Florence, Italy; 3 Unit of Oncological Gynecology, Centro di Riferimento Oncologico della Basilicata (IRCCS-CROB), Rionero in Vulture, Italy; 4 Advanced Biomedical Sciences Department, Naples Federico II University, AOU “FedericoII”, Naples, Italy

**Keywords:** anxiety, Covid-19, depression, peritraumatic distress, physical symptoms

## Abstract

**Background:**

The Coronavirus Disease (COVID-19) has had a significant impact on healthcare organizations, leading to a reduction in screening. The pandemic period has caused important psychological repercussions in the most fragile patients.

**Objectives:**

This study aimed to assess the levels of depression, anxiety, peri-traumatic stress, and physical symptoms in patients undergoing colposcopy during the COVID-19 pandemic and to compare these data with the post-pandemic period.

**Design:**

This longitudinal study included 96 individuals undergoing colposcopy, aged between 22 and 64, who were examined between March 2020 and December 2023.

**Methods:**

Participants were assessed at four distinct time points, referred to as T0, T1, T2, and T3. T0 encompassed the pandemic period, ranging from March 2020 to August 2020, while T1 occurred 1 year later, T2 and T3 correspond to data collected in 2022 and 2023. Statistical analysis was conducted to assess the impact of the COVID-19 pandemic on various psychological variables. Descriptive statistics, including means, standard deviations, and frequency distributions, were calculated for each psychological variable within each time period.

**Results:**

Our findings revealed a significant reduction in peri-traumatic stress and pain levels in the post-pandemic (from 2021 to 2023) period compared to the pandemic period. Conversely, anxiety and depression levels exhibited a statistically significant increase in the post-pandemic period and then gradually decrease in the subsequent follow-up.

**Conclusion:**

This study provides valuable insights into the profound impact of the COVID-19 pandemic on psychological distress experienced during the pandemic period itself, as well as its enduring effects on anxiety and depression in the subsequent period.

## Introduction

The Corona Virus Disease (COVID-19) health emergency has had a significant impact on prevention and screening activities. The National Screening Observatory has published a meticulous report on the first 8 months of 2020, which compares the data with that of 2019 and records a decrease in invitations and lower screening participation rates with a potential negative impact on mental health. Specifically, there was a noticeable 40% decrease in invitations and a drop of about 17% in the participation rate. Overall, an estimated 540,000 screenings were missed, leading to approximately 2400 missed diagnoses of precancerous lesions. While there was considerable regional variability, many services resumed in the second half of the year, and the delay gradually decreased.^1^

Prevention and screening in gynecology involve two levels of examination. The first level consists of a human papillomavirus (HPV) test and a Papanicolaou (Pap) test, offered to all women aged 25–64. The second level involves more targeted examinations, such as colposcopy, which are required when a positive result is obtained. The Pap test is administered to check for cervical neoplasia, as squamocolumnar junction cells of the endo- and ectocervix are susceptible to HPV infection and dysplastic changes. Unfortunately, HPV is responsible for more than 90% of cervical cancers.

Colposcopy specifically is an examination that involves observing the cervix through the colposcope, an optical instrument that allows 6- to 40-fold magnification. If necessary, colposcopy may be accompanied by cytological sampling (Pap test, HPV test) or targeted biopsy sampling performed under colposcopic guidance. This procedure is used to evaluate women with an abnormal Pap test, those positive for high-risk HPV DNA, or those with a suspicious cervix.^2,3^

Among the risks of the procedure, significant bleeding, infection, and long-term morbidity are low. Moreover, it is important to properly account for the patient’s anxiety and emotional distress as significant complications associated with the procedure. It can be challenging to determine whether negative feelings toward the procedure are related to the idea of HPV infection or to the procedure itself.^4^ Colposcopy is intended to help formulate a management plan based on the findings of biopsy pathology or the absence of findings.^5^ The waiting time for colposcopy can also have an impact on psychological distress.^6^

Abnormal Pap test results can evoke emotions in the patient as an outcome. Anxiety, involving excessive worry and fear about future uncertainties, and depression, characterized by persistent sadness and a loss of interest in daily activities, are the most common negative psychological outcomes, which can also cause significant distress for patients.^7,8^ Moreover, peri-traumatic stress refers to the stress experienced during or immediately after a traumatic event, such as a medical emergency or crisis, which can have lasting psychological effects.

This study aimed to assess the levels of anxiety, depression, peritraumatic stress, and other symptoms in patients treated during and after the COVID-19 pandemic period in four separate moments: during the pandemic and after 1, 2, and 3 years, respectively.

## Materials and methods

### Populations

In this cross-sectional study, 96 women aged 22–64 underwent to colposcopy from March 2020 to December 2023 (total of 46 months) were enrolled. Participants are selected randomly from the population, reducing bias. The inclusion criteria were aged between 20 and 65 years, abnormal cervical cytology and colposcopy examination at the Department of Gynecology and Psych-oncology of the IRCCS-CROB in Rionero in Vulture, Italy. No missing data were identified during the initial analysis and the preliminary analysis. The total sample comprised 96 patients of whom 100% were women, with an average age of 48.5 years (SD = 9.1, range: 22–63). The exclusion criteria comprised a history of psychological care or drug treatment for psychiatric disorders and diagnosis of cervical intraepithelial neoplasia or cancer. Before participating in the study, all participants were provided with detailed information about the study’s objectives and design and gave their written informed consent.

### Study design

During the study, participants were assessed at four different time points referred to as T0 (during pandemic period) and T1 (1 year later, 2021), T2 (2022) and T3 (2023). T0 took place between March 2020 and August 2020, during the pandemic. During this time, participants underwent a recruitment interview to gather clinical and anamnestic data. They also completed a set of standardized questionnaires such as Hospital Anxiety and Depression Scale (HADS), COVID-19 Peritraumatic Distress Index (CPDI), and Edmonton Symptom Assessment System (ESAS) to evaluate their levels of anxiety, depression, symptomatology, and peritraumatic stress (supplemental material). At T1, the same tests were repeated, and subsequently, patients underwent two follow-ups after 1 year and after 2 years (T2 and T3). The participants were asked to complete the questionnaire themselves while waiting for colposcopy, and it took approximately 30 min to fill out. All the tools are summarized in Table 1. The data were stored anonymously and organized to ensure easy access and security. This study was approved by the Local Ethics Committee, Comitato Etico Unico Regionale per la Basilicata, c/o Ufficio di Coordinamento della Segreteria T-S AOR San Carlo (protocol no. 1589) and was performed in accordance with the principles of the Declaration of Helsinki. The reporting of this study conforms to the STROBE statement.^9^

**Table 1.** Tools used for screening mental health disorders.

Tool	Items	Range	Total score
HADS	14 questions (7 for anxiety and 7 for depression)	4-point scale ranging from: 0 “not at all” to 3 “most of the time”	<7 normal 8–14 mild–moderate >14 severe
CPDI	24 items (anxiety, depression, specific phobias, cognitive change, avoidance and compulsive behavior, physical symptoms, and loss of social functioning)	5-point scale ranging from to 0 “not at all” to “4 extremely”	<28 normal distress 28–51 mild–moderate distress >51 severe distress
ESAS	9 items (pain, tiredness, drowsiness, nausea, lack of appetite, depression, anxiety, shortness of breath, and well-being)	11-point scale ranging from 0 “no symptom” to “10 worst possible symptom”	0–30 mild symptom 31–69 moderate symptom 70–100 severe symptom

HADS: Hospital Anxiety and Depression Scale; CPDI: COVID-19 Peritraumatic Distress Index; ESAS: Edmonton Symptom Assessment System.

#### Hospital Anxiety and Depression Scale

The HADS is a widely used self-report questionnaire that helps to assess symptoms of anxiety and depression in hospital settings. It has been validated in numerous languages and cultures. The Italian version of HADS consists of 14 questions, 7 related to anxiety and 7 related to depression.^10,11^

Each item is scored on a 4-point Likert scale that ranges from 0 (indicating “Not at all”) to 3 (indicating “Most of the time”). Using these scores, two separate subscales can be calculated: the anxiety subscale (HADS-A) and the depression subscale (HADS-D). This questionnaire has already been translated into Italian and used in clinical samples, demonstrating good psychometric properties (Cronbach’s alpha values between 0.80 and 0.85).^12^

#### The COVID-19 Peritraumatic Distress Index

The researchers utilized the CPDI to measure peritraumatic distress experienced during the pandemic.^13^ The CPDI is comprised of 24 items, and participants rated their response on a 5-point scale ranging from 0 (not at all) to 4 (extremely). The total score can range from 0 to 100, and score below 28 are indicative of normal distress, while score between 28 and 51 suggest mild-to-moderate distress and score above 51 indicate severe distress. The Italian version of the CPDI demonstrated strong psychometric properties with a Cronbach’s alpha value of 0.92.^14^

#### Edmonton Symptom Assessment System

ESAS is a tool originally designed for patients with advanced cancer, consisting of nine common symptoms (pain, fatigue, nausea, depression, anxiety, drowsiness, appetite, well-being, shortness of breath) with an additional specific symptom as the 10th question. It utilizes an 11-point scale ranging from 0 (no symptom) to 10 (worst possible symptom). After completing all the questions, the scores are summed up, and a total score of 0–30 indicates mild symptoms, 31–69 indicates moderate symptom, and 70–100 indicates severe symptoms.^15^

### Statistical analyses

Statistical analysis was conducted to assess the impact of the COVID-19 pandemic on various psychological variables, including peritraumatic stress, pain, weariness, nausea, drowsiness, breath, wellness, hunger, anxiety, and depression. Data were collected from a sample of participants across four distinct time periods: during, post-COVID-19 and 1 and 2 years after the end of pandemic

Descriptive statistics, including means (*M*), standard deviations (SD), and frequency distributions, were calculated for each psychological variable within each time period. These statistics provided an initial overview of the data and allowed for the examination of central tendencies and variability. Categorical variables were expressed as frequencies and percentages. If the variable was continuous, comparisons between the two groups (2019 versus 2020) were performed using the *t*-test; for categorical data, we used the χ^2^.

All significance tests were two-tailed using a significance level of α < 0.05. In order to determine the minimum sample size for the purpose of the study, we identified the depression as the end-point for all other end-points of the study. We tested the hypothesis that, from a mean basal value of the HADS questionnaires of 4.5, the impact of COVID will increase the point by at least 10% in the mean point. For this purpose, the study plan will include at least 190 patients (95 for questionnaire) with an estimated drop out of 15%–20%. It was calculated that a minimum of 156 evaluable patients (78 for questionnaire) should be enrolled with an α of 0.05 and a β of 0.80 (STATASOFt). Analyses were performed using the statistical package STATA, version 9.1.

## Results

Distress levels based on CPDI score, depression and anxiety levels based on HADS score, and pain score based on ESAS score in the inter population on measured four times (T0–T3) have been reported in the Table 2.

**Table 2.** Incidence of mental disorders during the four periods analyzed. Results.

Tool	Grade	T0 (*n* = 96)	T1 (*n* = 96)	T2 (*n* = 96)	T3 (*n* = 96)
CPDI, *n* (%)	Normal distress	60 (62.5)	64 (66.6)	95 (99)	96 (100)
	Mild–moderate distress	36 (37.5)	32 (33.4)	1 (1)	0 (0)
HADS anxiety, *n* (%)	Normal	77 (80.2)	51 (53.1)	75 (78.1)	91 (94.8)
	Mild–moderate	19 (19.8)	45 (46.9)	21 (21.9)	5 (5.2)
HADS depression, *n* (%)	Normal	91 (94.8)	66 (68.75)	86 (89.6)	91 (94.8)
	Mild–moderate	5 (5.2)	31 (31.25)	10 (10.4)	5 (5.2)
ESAS pain levels, *n* (%)	Normal	67 (69.8)	88 (91.7)	93 (90.6)	94 (97.9)
	Mild–moderate symptom	29 (30.2)	8 (8.3)	3 (9.4)	2 (2.1)

HADS: Hospital Anxiety and Depression Scale; CPDI: COVID-19 Peritraumatic Distress Index; ESAS: Edmonton Symptom Assessment System.

Notably, none of the patients reported severe levels of COVID-19 distress (scores >51), severe levels of depression (scores >14) and anxiety (scores >14), or severe levels of pain (scores >7).

There were no significant violations of assumptions, including normality, linearity, univariate or multivariate outliers, homogeneity of variance–covariance matrices, or multicollinearity during the preliminary assumption testing.

### COVID-19 Peritraumatic Distress Index

**Figure 1.**
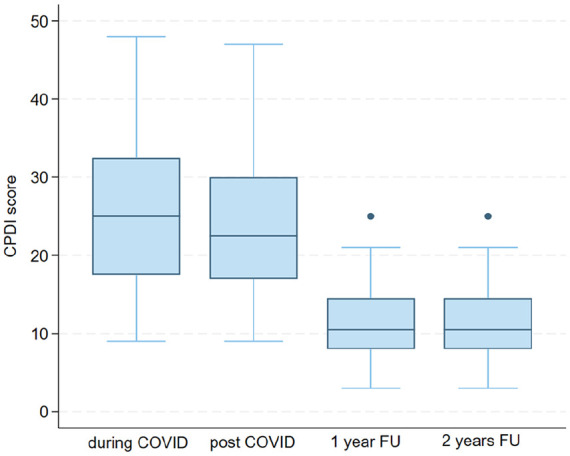
CPDI. CPDI: COVID-19 Peritraumatic Distress Index.

Statistically significant differences were observed in the CPDI pre- and at 1-year follow-up (FU) (mean = 18.11, SD = 10.03, *p* < 0.001) and CPDI pre- and at 2-year FU (mean = 18.11, SD = 10.03, *p* < 0.001). No differences were observed between CPDI pre and post-COVID-19 (24.22, SD = 8.93, *p* = 0.22) (Figure 1). Our research work, on the basis of the tests carried out, has seen a gradual decrease in the number of patients presenting mild–moderate peritraumatic stress which went from 36 (37.5% of the sample) during the pandemic to 32 in the first follow-up until to reach 1 at follow-up after 1 year and 0 after 2 years.

### Hospital Anxiety and Depression Scale

**Figure 2.**
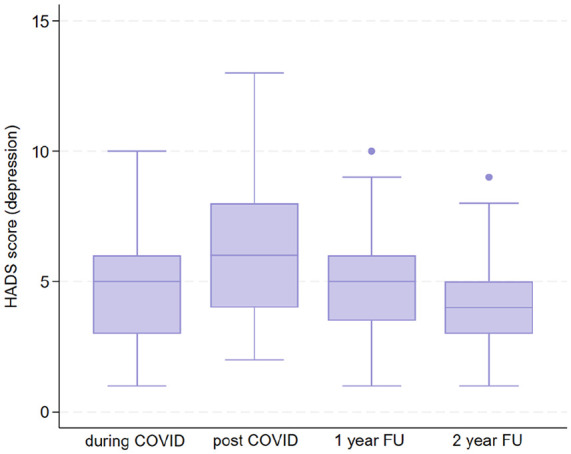
HADS depression. HADS: Hospital Anxiety and Depression Scale.

**Figure 3.**
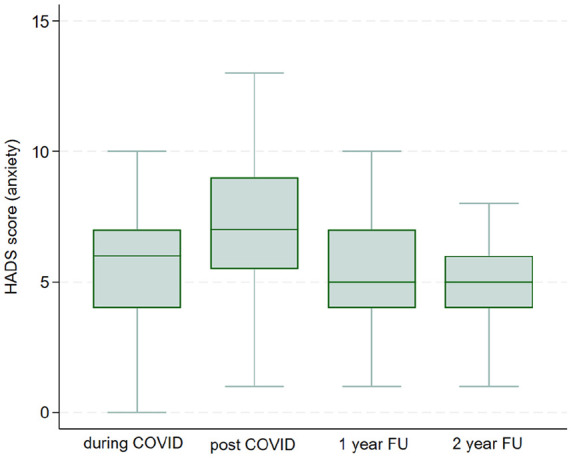
HADS anxiety. HADS: Hospital Anxiety and Depression Scale.

Regarding HADS depression score, a statistically significant differences were observed between pre- and post-COVID-19 pandemic (mean = 5.36, SD = 2.48, *p* < 0.001), while any difference was observed between pre- and at 1-year FU (mean = 4.72, SD = 1.95, *p* = 0.19), and between pre- and at 2-year FU (mean = 4.42, SD = 1.80, *p* = 0.38) (Figure 2). Regarding HADS anxiety, pre- and post-COVID-19 (mean = 6.44, SD = 2.55, *p* < 0.001), and pre- and at 2-year FU (mean = 5.16, SD = 1.95, *p* = 0.042), a statistically differences was observed, conversely to pre- and at 1-year FU (mean = 5.57, SD = 2.10, *p* = 0.91) (Figure 3). However, the data regarding anxiety and depression are vastly different. There are 19 patients with mild-to-moderate anxiety levels during the pandemic, which rose to 45 at T1, and then gradually dropped to 21 at T2 and to 5 at T3. Regarding data on depression, there are 5 patients with mild-to-moderate symptoms during the pandemic, they rise to 31 at T1 and drops again in T2 (10 patients) and in T3 (5 patients).

### Edmonton Symptom Assessment System

**Figure 4.**
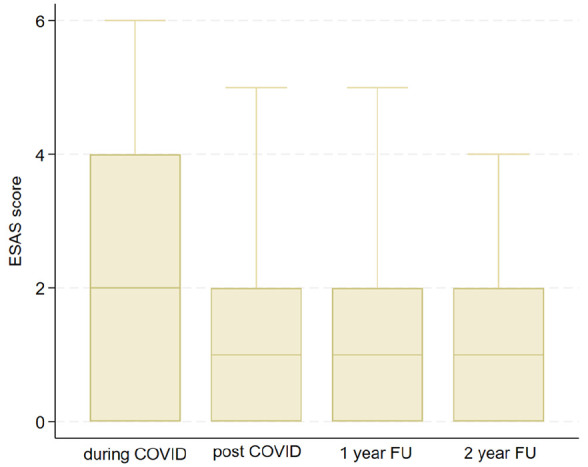
ESAS pain. ESAS: Edmonton Symptom Assessment System.

Regarding ESAS pain score, statistically significant differences were observed between pre- and post-COVID-19 pandemic (mean = 1.72, SD = 1.55, *p* = 0.017), between pre- and at 1-year FU (mean = 1.56, SD = 1.54, *p* < 0.001), and between pre- and at 2-year FU (mean = 1.48, SD = 1.50, *p* < 0.001) (Figure 4).

The values regarding pain have approximately the same trend, starting from 29 patients with mild-to-moderate pain to 8, 3, and 2 patients, respectively, in the 3 subsequent follow-ups. The complete data are summarized in Table 2.

## Discussion

Screening assessments represent a valuable preventive strategy aimed at reducing cancer occurrence and subsequent mortality rates.^16^ While prevention and screening play a crucial role in both individual and public health,^17^ the landscape of cancer healthcare has undergone substantial transformations due to the impact of the COVID-19 pandemic.^18^ The COVID-19 pandemic has had a pronounced impact on individuals’ lifestyles and behaviors, resulting in widespread alterations in the realms of health, socioeconomic factors, and psychology.^19,20^ The swift proliferation of the virus and its mutations indeed constituted a distressing event for the general populace, particularly individuals with preexisting medical conditions. Over the past 2 years, there has been a substantial increase in evidence concerning the psychological repercussions of the virus on the general population.^21,22^ Nevertheless, available data on the psychological effects of the COVID-19 pandemic on individuals with cancer are both limited and diverse.^23–27^

Despite the current shortage of literature on this topic, it does indicate that the conditions imposed by the COVID-19 pandemic have led to elevated levels of anxiety and depressive symptoms among cancer patients.^21,24^

The pandemic caused by SARS-CoV-2 has significantly altered the organization of healthcare systems worldwide. A primary objective of public health during the COVID-19 pandemic was to mitigate the strain on healthcare systems. In Italy, during the initial phase of the pandemic (commencing from March 16, 2020), all outpatient services were postponed,^1^ including the HPV vaccination program, cervical cancer screening, colposcopy clinic activities, and outpatient surgery of the lower genital tract, except for cases necessitating urgent evaluation.^28^

In response to these challenges, scientific societies issued guidance to assist clinicians and service providers in triaging patients requiring prompt evaluation in a colposcopy clinic from those whose care could be safely deferred.^29,30^

This study aimed to investigate peritraumatic distress, anxiety, depression, and physical symptoms in a female population undergoing colposcopy during the COVID-19 pandemic and during three subsequent follow-up periods. The research hypothesis posited that patients would report higher levels of anxiety and depression after the pandemic compared to the during-pandemic period, and that COVID-19 peritraumatic distress would significantly impact these variables. Our findings aligned with this hypothesis, indicating a significant reduction in peritraumatic stress and pain levels after the pandemic compared to the pandemic period in each follow-up. Specifically, female gynecological patients in our sample reported notably lower scores for peritraumatic stress and physical pain post-pandemic and in two subsequent follow-up periods compared to their baseline measurement: CPDI pre- and at 1-year FU (*p* < 0.001) and CPDI pre- and at 2-year FU (*p* < 0.001); ESAS pain score, pre- and post-COVID-19 pandemic (*p* = 0.017), between pre- and at 1-year FU (*p* < 0.001), and between pre- and at 2-year FU (*p* < 0.001).

However, we did not observe significant changes in fatigue, nausea, drowsiness, breath, wellness, or hunger. Anxiety and depression levels exhibited a significant increase in the post-pandemic period, likely attributed to concerns about the accessibility of screening procedures during potential lockdown scenarios. HADS anxiety and depression are statistically significant: HADS anxiety, pre- and post-COVID-19 (*p* < 0.001) and pre- and at 2-year FU (*p* = 0.042). HADS depression score are between pre- and post-COVID-19 pandemic (*p* < 0.001).

Moreover, the pandemic period appeared to exacerbate stress conditions and accentuate previously minor psychological issues.

Our research experience revealed an increase in anxiety and depressive symptoms in the post-COVID period, which diminished in the two follow-up periods after 1 and 2 years. Nevertheless, it is essential to acknowledge that over such an extended period, numerous additional factors may have occurred, making them difficult to evaluate.

These findings suggest that the pandemic contributed to precipitating psychological distress closely linked to the COVID-19 pandemic, with lasting psychological repercussions in the subsequent period.

### Limitations

Our study has limitations that must be taken into consideration. First, the sample size is relatively small; second, we reported the experience of a single cancer center and a single region of Italy. It may be appropriate in the future to conduct studies involving several centers in different geographic areas and with larger samples to compare the results obtained. Additionally, there was no control group whose results could be compared with those of the sample of cancer patients that we analyzed. Therefore, future studies should also include a group of healthy subjects to improve further the significance of the results obtained.

However, to the best of our knowledge, this study represents the only investigation into the development and extent of several significant mental health disorders among women undergoing cervical cancer screening during the pandemic.

## Conclusions

Our study has provided valuable insights into the substantial impact of the COVID-19 pandemic on distress during the pandemic itself, as well as its enduring effects on anxiety and depression in the subsequent period. Consistent with our hypothesis, it emerged that peritraumatic stress and pain decreased as we moved away from the pandemic period. However, the data regarding anxiety and depression show a different trend, indicating an increase in the period immediately following the pandemic, followed by a gradual decrease in the subsequent 2 years. We believe this is justified by the fact that the perception of danger is contextualized and therefore greater in the emergency phase. Regarding the increase in post-pandemic anxiety and depression values, it appears related not only to the fear of not being able to receive adequate treatments in a timely manner but also to an increase in the general population.

Further research efforts will undoubtedly be indispensable to further elucidate the risk and protective factors associated with the psychological repercussions of the pandemic, particularly within specific demographic groups.

## Supplemental Material

sj-docx-1-whe-10.1177_17455057241308342 – Supplemental material for Evaluation of mental disorder related to colposcopy procedure during the COVID period: A cross-sectional study Supplemental material, sj-docx-1-whe-10.1177_17455057241308342 for Evaluation of mental disorder related to colposcopy procedure during the COVID period: A cross-sectional study by Ilaria Bochicchio, Martina Catalano, Giovanni Deiana, Giandomenico Roviello, Pasquale Marino, Fabrizia Calenda, Alessandro R Lettini and Francesca Sanseverino in Women’s Health

sj-docx-2-whe-10.1177_17455057241308342 – Supplemental material for Evaluation of mental disorder related to colposcopy procedure during the COVID period: A cross-sectional study Supplemental material, sj-docx-2-whe-10.1177_17455057241308342 for Evaluation of mental disorder related to colposcopy procedure during the COVID period: A cross-sectional study by Ilaria Bochicchio, Martina Catalano, Giovanni Deiana, Giandomenico Roviello, Pasquale Marino, Fabrizia Calenda, Alessandro R Lettini and Francesca Sanseverino in Women’s Health

sj-docx-3-whe-10.1177_17455057241308342 – Supplemental material for Evaluation of mental disorder related to colposcopy procedure during the COVID period: A cross-sectional study Supplemental material, sj-docx-3-whe-10.1177_17455057241308342 for Evaluation of mental disorder related to colposcopy procedure during the COVID period: A cross-sectional study by Ilaria Bochicchio, Martina Catalano, Giovanni Deiana, Giandomenico Roviello, Pasquale Marino, Fabrizia Calenda, Alessandro R Lettini and Francesca Sanseverino in Women’s Health

## Declarations

**Ethics approval and consent to participate:** This study protocol was reviewed and approved by Comitato Etico Unico Regionale per la Basilicata, c/o Ufficio di Coordinamento della Segreteria T-S AOR San Carlo, approval number 1589, July 3, 2023 name of ethic committee: Comitato Etico Unico Regionale per la Basilicata, c/o Ufficio di Coordinamento della Segreteria T-S AOR San Carlo, approval number 1589, July 3, 2023.

**Consent to participate statement:** Written informed consent was obtained from all subjects involved in the study.

**Consent to publication:** Written informed consent was obtained from all subjects involved in the study.

**Availability of data and materials:** The data presented in this study are available on request from the corresponding author. The data are not publicly available to ensure the privacy of the patients participating in the study.
