# Supplementary material for: Evaluation of mental disorder related to colposcopy procedure during the COVID period: A cross-sectional study
Source: Womens Health (Lond). 2025 Jan 18;21:17455057241308342. doi: 10.1177/17455057241308342 (PMC11742171; doi:10.1177/17455057241308342)
Supplement: sj-docx-1-whe-10.1177_17455057241308342 – Supplemental material for Evaluation of mental disorder related to colposcopy procedure during the COVID period: A cross-sectional study [file sj-docx-1-whe-10.1177_17455057241308342.docx]

**The Covid-19 Peritraumatic Distress Index (CPDI) in English**

Please select the frequency of the below activities in the last week.

|  | Never | Occasionally | Sometimes | Often | Most  of the  time |
| --- | --- | --- | --- | --- | --- |
| 1. Compared to usual, I feel more nervous and anxious | 0 | 1 | 2 | 3 | 4 |
| 2. I feel insecure and bought a lot of goods, such as medications,  sanitizer, gloves, masks, and/or other home supplies | 0 | 1 | 2 | 3 | 4 |
| 3. I can’t stop myself from imagining myself or my family being  infected and feel terrified and anxious about it | 0 | 1 | 2 | 3 | 4 |
| 4. I feel empty and helpless no matter what I do | 0 | 1 | 2 | 3 | 4 |
| 5. I feel sympathetic to the Covid-19 patients and their families. I feel sad about them. | 0 | 1 | 2 | 3 | 4 |
| 6. I feel helpless and angry about people around me, such as the  governors and media | 0 | 1 | 2 | 3 | 4 |
| 7. I am losing faith in the people around me | 0 | 1 | 2 | 3 | 4 |
| 8. I collect information about Covid-19 all day. Even if it’s not necessary, I can’t stop myself | 0 | 1 | 2 | 3 | 4 |
| 9. I will believe the Covid-19 information from all sources without any evaluation | 0 | 1 | 2 | 3 | 4 |
| 10. I would rather believe in negative news about Covid-19 and be skeptical about the good news | 0 | 1 | 2 | 3 | 4 |
| 11. I am constantly sharing news about Covid-19 (mostly negative news) | 0 | 1 | 2 | 3 | 4 |
| 12. I avoid watching Covid-19 news, since I am too scared to do so | 0 | 1 | 2 | 3 | 4 |
| 13. I am more irritable and have frequent conflicts with my family | 0 | 1 | 2 | 3 | 4 |
| 14. I feel tired and sometimes even exhausted | 0 | 1 | 2 | 3 | 4 |
| 15. Due to feelings of anxiety, my reactions are becoming sluggish. | 0 | 1 | 2 | 3 | 4 |
| 16. I find it hard to concentrate | 0 | 1 | 2 | 3 | 4 |
| 17. I find it hard to make any decisions | 0 | 1 | 2 | 3 | 4 |
| 18. During this Covid-19 outbreak, I often feel dizzy, have back pain, or chest discomfort | 0 | 1 | 2 | 3 | 4 |
| 19. During this Covid-19 outbreak, I often feel stomach pain, bloating, or other stomach discomforts | 0 | 1 | 2 | 3 | 4 |
| 20. I feel uncomfortable when communicating with others | 0 | 1 | 2 | 3 | 4 |
| 21. Recently, I rarely talk to my family | 0 | 1 | 2 | 3 | 4 |
| 22. I cannot sleep well. I always dream about myself or my family being infected by coronavirus | 0 | 1 | 2 | 3 | 4 |
| 23.I lost my appetite | 0 | 1 | 2 | 3 | 4 |
| 24. I have constipation or frequent urination | 0 | 1 | 2 | 3 | 4 |

**Note**: CPDI = sum of the score of each question +4.

CPDI> 52: severe distress; CPDI within 28-51: mild/moderate distress; CPDI< 28: Normal;
