# Supplementary material for: Evaluation of mental disorder related to colposcopy procedure during the COVID period: A cross-sectional study
Source: Womens Health (Lond). 2025 Jan 18;21:17455057241308342. doi: 10.1177/17455057241308342 (PMC11742171; doi:10.1177/17455057241308342)
Supplement: sj-docx-2-whe-10.1177_17455057241308342 – Supplemental material for Evaluation of mental disorder related to colposcopy procedure during the COVID period: A cross-sectional study [file sj-docx-2-whe-10.1177_17455057241308342.docx]

Edmonton Symptom Assessment System Revised (ESAS-r)

Please circle the number that best describes how you feel NOW:

| No Pain | 0 | 1 | 2 | 3 | 4 | 5 | 6 | 7 | 8 | 9 | 10 | Worst Possible |
| --- | --- | --- | --- | --- | --- | --- | --- | --- | --- | --- | --- | --- |
|  |  |  |  |  |  |  |  |  |  |  |  | Pain |
| No Tiredness | 0 | 1 | 2 | 3 | 4 | 5 | 6 | 7 | 8 | 9 | 10 | Worst Possible |
| *(Tiredness = lack of energy)* Tiredness | | | | | | | | | | | | |
| No Drowsiness | 0 | 1 | 2 | 3 | 4 | 5 | 6 | 7 | 8 | 9 | 10 | Worst Possible |
| *(Drowsiness = feeling sleepy)* Drowsiness | | | | | | | | | | | | |
| No Nausea | 0 | 1 | 2 | 3 | 4 | 5 | 6 | 7 | 8 | 9 | 10 | Worst Possible |
|  |  |  |  |  |  |  |  |  |  |  |  | Nausea |
| No Lack of Appetite | 0 | 1 | 2 | 3 | 4 | 5 | 6 | 7 | 8 | 9 | 10 | Worst Possible |
|  |  |  |  |  |  |  |  |  |  |  |  | Lack of Appetitie |
| No Shortness of | 0 | 1 | 2 | 3 | 4 | 5 | 6 | 7 | 8 | 9 | 10 | Worst Possible |
| Breath |  |  |  |  |  |  |  |  |  |  |  | Shortness of Breath |
| No Depression | 0 | 1 | 2 | 3 | 4 | 5 | 6 | 7 | 8 | 9 | 10 | Worst Possible |
| *(Depression = feeling sad)* |  |  |  |  |  |  |  |  |  |  |  | Depression |
| No Anxiety | 0 | 1 | 2 | 3 | 4 | 5 | 6 | 7 | 8 | 9 | 10 | Worst Possible |
| *(Anxiety = feeling nervous)* |  |  |  |  |  |  |  |  |  |  |  | Anxiety |
| Best Wellbeing | 0 | 1 | 2 | 3 | 4 | 5 | 6 | 7 | 8 | 9 | 10 | Worst Possible |
| *(Wellbeing = how you feel overall)* Wellbeing | | | | | | | | | | | | |
| No | 0 | 1 | 2 | 3 | 4 | 5 | 6 | 7 | 8 | 9 | 10 | Worst Possible |
| Other Problem *(For example constipation)* | | | | | | | | | | | | |

| Patient Name | Completed by *(Check one)*   - Patient - Family Caregiver - Health Care Professional Caregiver - Caregiver-assisted |
| --- | --- |
| Date *(yyyy-Mon-dd)* |  |
| Time *(hh:mm)* |  |

07903(Rev2015-08)
